# Trimeric Form of Intracellular ATP Synthase Subunit β of *Aggregatibacter actinomycetemcomitans* Binds Human Interleukin-1β

**DOI:** 10.1371/journal.pone.0018929

**Published:** 2011-04-18

**Authors:** Annamari Paino, Heidi Tuominen, Mari Jääskeläinen, Jonna Alanko, Jari Nuutila, Sirkka E. Asikainen, Lauri J. Pelliniemi, Marja T. Pöllänen, Casey Chen, Riikka Ihalin

**Affiliations:** 1 Department of Biochemistry and Food Chemistry, University of Turku, Turku, Finland; 2 Oral Microbiology, Department of Odontology, Umeå University, Umeå, Sweden; 3 Laboratory of Electron Microscopy, University of Turku, Turku, Finland; 4 Department of Periodontology, Institute of Dentistry, University of Turku, Turku, Finland; 5 Division of Periodontology, Diagnostic Sciences & Dental Hygiene, USC School of Dentistry, University of Southern California, Los Angeles, California, United States of America; University of Birmingham, United Kingdom

## Abstract

Bacterial biofilms resist host defenses and antibiotics partly because of their decreased metabolism. Some bacteria use proinflammatory cytokines, such as interleukin (IL)-1β, as cues to promote biofilm formation and to alter virulence. Although one potential bacterial IL-1β receptor has been identified, current knowledge of the bacterial IL-1β sensing mechanism is limited. In chronic biofilm infection, periodontitis, *Aggregatibacter actinomycetemcomitans* requires tight adherence (*tad*)-locus to form biofilms, and tissue destroying active lesions contain more IL-1β than inactive ones. The effect of IL-1β on the metabolic activity of *A. actinomycetemcomitans* biofilm was tested using alamarBlue™. The binding of IL-1β to *A. actinomycetemcomitans* cells was investigated using transmission electron microscopy and flow cytometry. To identify the proteins which interacted with IL-1β, different protein fractions from *A. actinomycetemcomitans* were run in native-PAGE and blotted using biotinylated IL-1β and avidin-HRP, and identified using mass spectroscopy. We show that although IL-1β slightly increases the biofilm formation of *A. actinomycetemcomitans*, it reduces the metabolic activity of the biofilm. A similar reduction was observed with all *tad*-locus mutants except the secretin mutant, although all tested mutant strains as well as wild type strains bound IL-1β. Our results suggest that IL-1β might be transported into the *A. actinomycetemcomitans* cells, and the trimeric form of intracellular ATP synthase subunit β interacted with IL-1β, possibly explaining the decreased metabolic activity. Because ATP synthase is highly conserved, it might universally enhance biofilm resistance to host defense by binding IL-1β during inflammation.

## Introduction

Bacterial biofilms are resistant to host defense factors and antibiotics because of their protective extracellular matrix and dormant persister cells [1], [2]. However, hiding and hibernating might not be the only strategies that biofilm cells use to thwart host defenses. *In vitro* studies with *Staphylococcus aureus* suggest that biofilm-forming bacterial cells may sense and respond to inflammation of the host by binding proinflammatory cytokines, thereby leading to enhanced biofilm formation [3] and altered virulence [4]. *S. aureus* biofilm cells bind more interleukin (IL)-1β than the respective planktonic cells [3], and IL-1β increases the expression levels of virulence-associated adhesion molecules and decreases the expression levels of leukotoxins of *S. aureus* [4]. It has been suggested that bacterial cells might internalize IL-1β to alter their gene expression pattern [4]. However, little attention has been focused on investigating the bacterial molecular mechanism that senses proinflammatory cytokines, especially IL-1β.

Periodontitis is a chronic infection caused by bacterial biofilms. In addition to causing local tissue destruction, periodontitis also elevates circulating levels of inflammatory mediators [5]–[8], which may contribute to the development of systemic diseases such as cardiovascular diseases [9]. In periodontal inflammation and tissue destruction, there is an alternation between active and less active phases, and the active phase is often associated with an increased production of IL-1β [10], suggesting pathogen-host crosstalk. The crosstalk between *Actinobacillus pleuropneumoniae* cells and host epithelial cells has been shown to cause an upregulation of biofilm-associated bacterial genes, including secretin coding *rcpA* [11]. A member of the same *Pasteurellaceae* family, *Aggregatibacter (Actinobacillus) actinomycetemcomitans*, is related with aggressive forms of periodontitis. *In vitro A. actinomycetemcomitans* forms tight biofilms using a fimbriae network, and in the rat model of periodontitis, a genetic locus *tad* is responsible for fimbriae biogenesis and adherence [12], making it a significant virulence factor [13]. The *tad* gene cluster has been identified in various gram-negative and gram-positive species, including well known human pathogens such as *Bordetella pertussis*, *Mycobacterium tuberculosis*, *Pseudomonas aeruginosa* and *Vibrio cholerae* [12]. The *A. actinomycetemcomitans tad* locus codes for 14 different proteins (Flp1-Flp2-TadV-RcpCAB-TadZABCDEFG), of which 13, excluding the pseudopilin (Flp2), form the macromolecular machinery for fimbriae biogenesis [14]. Four of these proteins (RcpA, RcpB, RcpC, and TadD) are located on the outer membrane of *A. actinomycetemcomitans* [15], of which RcpA belongs to the protein superfamily of secretins [15]. The function of RcpA is to form a channel through which the Flp1 pili are secreted outside the outer membrane [12], [16]. Although the main function of secretins is to secrete proteins across the outer membrane of Gram-negative species [17], some of them, such as PilQ of *Neisseria meningitides*, have been argued to also have an opposite role. The *N. meningitides* PilQ multimer has been proposed to function as an entrance channel for DNA in naturally competent cells [18].

Although the bacterial-host interaction has been shown to upregulate bacterial genes crucial to biofilm formation [11], and IL-1β changes the virulence gene expression pattern of *S. aureus* [4], little is known about the bacterial machinery that senses the inflammatory milieu of the host. Because IL-1β is produced in the active phase of periodontitis [10], the aim of this study was to investigate the effects of IL-1β on the formation and the metabolic activity of the biofilm of a periodontal pathogen, *A. actinomycetemcomitans*, and identify bacterial proteins that interact with IL-1β. In addition, the possible role of *tad*-locus outer membrane proteins in interactions between IL-1β and *A. actinomycetemcomitans* was studied using single gene deletion mutants. The results showed that IL-1β bound and entered into the *A. actinomycetemcomitans* cells and decreased their metabolic activity. Additionally, the *tad* locus secretin, RcpA, might have a role in the IL-1β internalization process. The trimeric form of a conserved intracellular protein, ATP synthase subunit β, involved in energy production, interacted with IL-1β. Although the interaction can not be claimed to be specific for IL-1β, it might explain the decrease in metabolic activity.

## Results

### Formation of A. actinomycetemcomitans biofilm in the presence of human IL-1β

**Figure 1:**
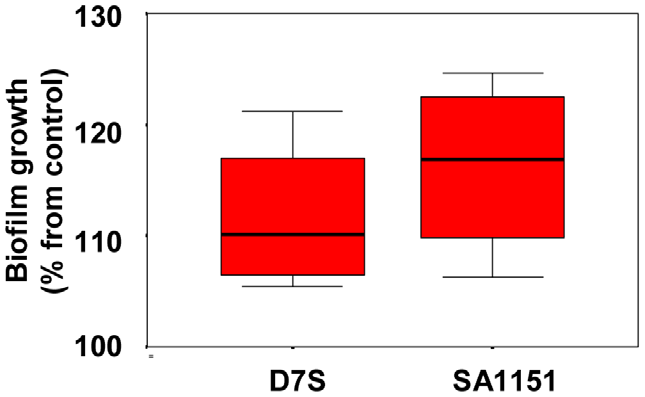
Effect of IL-1β on the formation of *A. actinomycetemcomitans* biofilm. The tested strains D7S (serotype a) and SA1151 (serotype c) were rough-colony forming clinical isolates, which produced fimbriae. Pre-grown (approximately 18 h) biofilms were grown with/without IL-1β (10 ng/ml) in RPMI 1640 medium, and the formation of biofilm was estimated with crystal violet staining after 6 h incubation. The box-plot represents data from four independent experiments.

Human recombinant IL-1β (10 ng/ml) increased statistically significantly (p<0.05; Mann-Whitney U-test) the quantity of measurable biofilm mass of both tested *A. actinomycetemcomitans* strains D7S (serotype a) and SA1151 (serotype c) after 6 h incubation as compared to the control incubation without IL-1β (Fig. 1).

### Metabolic activity of IL-1β treated *A. actinomycetemcomitans* cells

**Figure 2:**
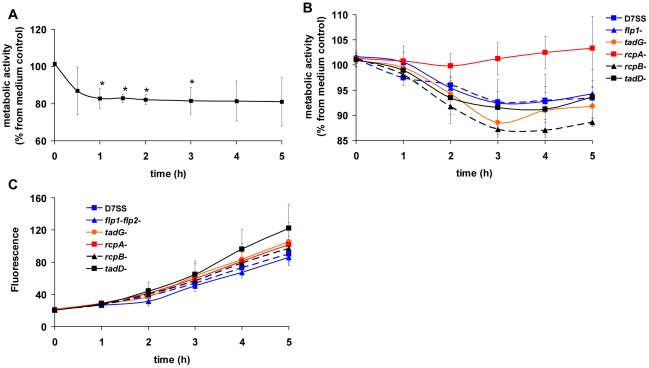
Effect of IL-1β on the metabolic activity of *A. actinomycetemcomitans*. The tested strains D7S (serotype a) was rough colony forming isolate, which produced fimbriae. Pre-grown (approximately 18 h) biofilms were grown with human IL-1β (10 ng/ml) or without (control) in RPMI 1640 medium and the metabolic activity was followed with alamarBlue™ (Panel A). Data is shown as percentage of reduced alamarBlue compared to control. The spontaneous smooth-colony variant (D7SS) of D7S and its planktonic single-gene deletion mutants *rcpA*, *rcpB*, *tadD*, *tadG*, and *flp1-flp2* were studied (Panel B and C). The pre-grown (18 h) cells were incubated with or without human IL-1β (10 ng/ml) in RPMI 1640 medium, and the metabolic activity was followed using alamarBlue™. The results are shown as percentage of metabolic activities of IL-1β-containing reactions compared to control cultures (Panel B). The metabolic activities of planktonic control cultures (sterile water was substituted for IL-1β) are presented as the fluorescence of the reduced form of alamarBlue™ (Panel C). All results are shown as means ± SD from three independent experiments. Each experiment contained duplications of each reaction.

The metabolic activity of biofilm from rough colony *A. actinomycetemcomitans* isolate D7S decreased when incubated with human IL-1β (Fig. 2A). Statistically significant decrease (p<0.05, T-test) was observed after one hour and it lasted for two hours (Fig. 2A). The decrease in metabolic activity was transient with clinical strains SA1398 and SA1151 (data not shown). The phenomenon seemed to be dependent on the IL-1β/cell ratio. When lower numbers of cells were used, the drop in metabolic activity of strains SA1398 and SA1151 was similar as detected with strain D7S (data not shown).

The metabolic activity of planktonic cells of *A. actinomycetemcomitans* decreased when incubated with human IL-1β when the genome contained the intact *rcpA* gene (Fig. 2B). However, when *rcpA* was deleted, the D7S strain became unresponsive to IL-1β (Fig. 2B, red line). All planktonic strains showed a similar curve for alamarBlue™ reduction over time in the absence of IL-1β (Fig. 2C), so the decreased metabolic activity observed in the Δ*rcpA* mutant was not due to defective metabolism.

### IL-1β internalization

**Figure 3:**
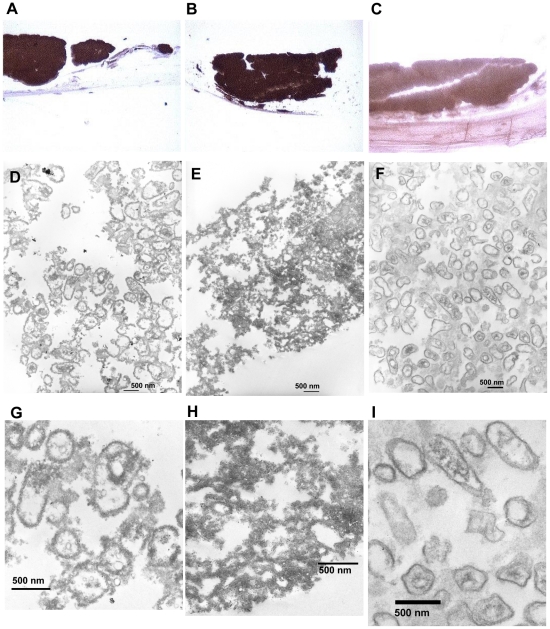
Internalization of IL-1β by *A. actinomycetemcomitans* co-cultured with organotypic oral mucosa. Formalin fixed paraffin sections of *A. actinomycetemcomitans* biofilm containing co-cultures were treated with anti-IL-1β (Panel A), anti-N-terminal-RcpA (Panel B), or control IgG (Panel C) after which the binding antibodies were detected with the NovoLink™ Polymer Detection System (Novocastra™). The sections with the DAB-label were stained with osmium for electron microscopy. Anti-IL-1β stained samples showed structures of *A. actinomycetemcomitans* cell shape and size (Panel D), with dark precipitate in both extra- and intracellular space (Panel G). The anti-RcpA stained positive control showed intense staining (Panel B) with similar structures (Panel E), although the cell structures are less visible due to the extent of the extracellular precipitate (Panel H). Control IgG antibody showed less staining (Panel C) revealing similar structures (Panel F) without dark precipitates bound to the cell membranes (Panel I).

*A. actinomycetemcomitans* D7S biofilm bound IL-1β when co-cultured with organotypic oral mucosa (Fig. 3A). The anti-IL-1β stained samples of organotypic oral mucosa–*A. actinomycetemcomitans* biofilm co-culture showed structures similar to the size and shape of *A. actinomycetemcomitans* cells (Fig. 3D and G). In these structures, dark precipitates were located both out- and inside of the bacterial cells (Fig. 3G). Similar structures were observed in anti-RcpA stained samples (3E and H), although the extent of precipitate in the extracellular space prevented the clear detection of the structures (Fig. 3H). The isotype control IgG stained samples showed significantly less intense staining (Fig. 3C) than the anti-IL-1β stained samples, and the bacterial structures were clearer defined (3F and I) without any detectable dark precipitates (Fig. 3I).

### Binding of biotinylated IL-1β on whole cells and different protein fractions of *A. actinomycetemcomitans*

**Figure 4:**
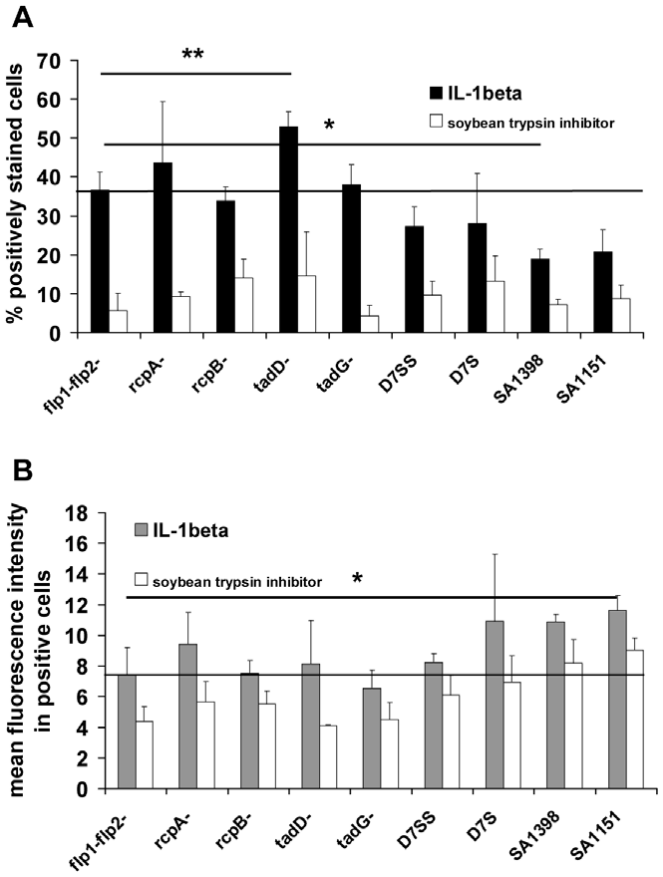
Binding of IL-1β on *A. actinomycetemcomitans* cells. The binding on fixed *A. actinomycetemcomitans* cells was studied using flow cytometry. The cells were first treated with biotinylated IL-1β after which they were stained with avidin-FITC. Panel A presents the percentage of positively stained cells as detected with flow cytometer. Panel B shows the mean fluorescence intensity in positively stained cells. Results are shown as means + SD of three independent experiments. Statistically significant (Two sample paired T-test) differences between the test strain and Δ*flp1-flp2* mutant (flp1-flp2-) are marked with * and **, indicating p≤0.05 and p≤0.01, respectively. Solid line indicates the level obtained with the Δ*flp1-flp2* mutant. Biotinylated soybean trypsin inhibitor was used as a negative control protein.

Biotinylated IL-1β bound more efficiently than biotinylated soybean trypsin inhibitor (control protein) on whole cells of all *A. actinomycetemcomitans* strains, whether biofilm forming or planktonic (p<0.001; Wilcoxon Signed Ranks Test) (Fig. 4A). Because deletion of any of the proteins did not decrease the binding significantly (p>0.05; Wilcoxon Signed Ranks Test), none of the deletion mutant proteins coded by *tad*-locus genes was responsible for the specific binding of IL-1β. Only the spontaneous smooth-colony variant (D7SS) of D7S showed a slight decrease in the percentage of IL-1β positive cells (Fig. 4A), but this decrease was not statistically significant. However, the *ΔtadD* mutant showed much more binding than the *Δflp1-flp2* mutant (p = 0.007; Paired samples T-test), of which the latter expressed intact machinery for fimbriae production. In addition, IL-1β bound slightly better on the *ΔrcpA* mutant than on *Δflp1-flp2* mutant cells, but the difference between these mutants was not statistically significant. All fimbriated biofilm-forming isolates (D7S, SA1398, SA1151) bound less IL-1β than the *Δflp1-flp2* mutant when the percentages of positively stained cells were compared, although only with strain SA1398 was the difference statistically significant (p = 0.046; Paired samples T-test) (Fig. 4A). However, although the mean fluorescence intensity (*i.e.*, the amount of cell-bound biotinylated IL-1β per individual positively stained cell) was somewhat higher with biofilm-forming isolates (D7S, SA1398, SA1151) than with the *Δflp1-flp2* mutant (Fig. 4B), only SA1151 showed a statistically significant difference (p = 0.014; Paired samples T-test). Similar results were also observed with the biotinylated control protein (Fig. 4B).

**Figure 5:**
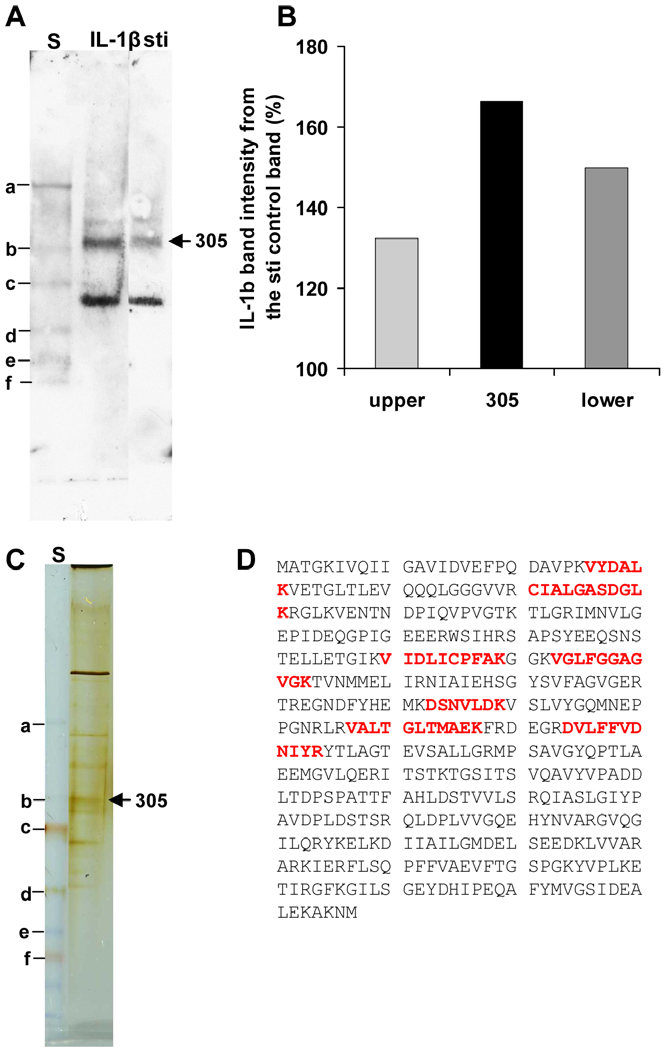
Binding of IL-1β to intracellular protein fraction of *A. actinomycetemcomitans*. The samples were run in native-PAGE, and the proteins were transferred to nitrocellulose membrane. The reactive bands were then detected using biotinylated IL-1β and, HRP-linked streptavidin. Control detection included biotinylated soybean trypsin inhibitor (sti). Reactive protein band 305 (named according to its travelling distance in these gels), which was more pronounced in IL-1β incubated detection than in the control detection, was isolated from identical gel after silver staining. The isolated protein band 305 was digested with trypsin before peptide separation and peptide identification with LC-MS/MS. Panel A shows the blots treated with either IL-1β or sti. Panel B shows the intensities of the three reactive bands in IL-1β-treated membranes compared to sti-treated membranes. Panel C presents the silver stained native-PAGE gel. Panel D shows the seven peptides that were identified from protein band 305 (denoted by the sequences given in bold type). According to the peptide sequence data, the protein was identified as ATP synthase subunit β (*Aggregatibacter actinomycetemcomitans* D11S-1) [42]. The Mascot program combined with Sprot-Trembl (uniprot) protein sequence database was used to the protein identification. The gels in Panels A and B contained PageRuler™ Plus Prestained Protein Ladder (denoted by “S”), and some of the proteins in the ladder are denoted (a–f).

To further characterize the binding of IL-1β on *A. actinomycetemcomitans* cells, soluble protein fractions were extracted from the cells and their IL-1β binding capacity was determined. Of the four fractions tested, only the intracellular protein fraction, which was soluble in 10 mM HEPES at pH 7.4, was bound to biotinylated recombinant IL-1β when detected by blotting after native-PAGE. Three protein bands interacted with biotinylated IL-1β (Fig. 5A). A reactive protein band, named 305 according to its traveling distance in native-PAGE, bound more effectively to IL-1β than control protein, as it was not detected as a prominent band in the control membrane (Fig. 5B).

### Identification of IL-1β binding protein and characterization of the binding

Protein band 305 was extracted from the silver stained native-PAGE gel (Fig. 5C) and identified as ATP synthase subunit β by LS/ESI-MS/MS analysis. Seven peptides, covering 14% of the sequence (Fig. 5D), were identified.

**Figure 6:**
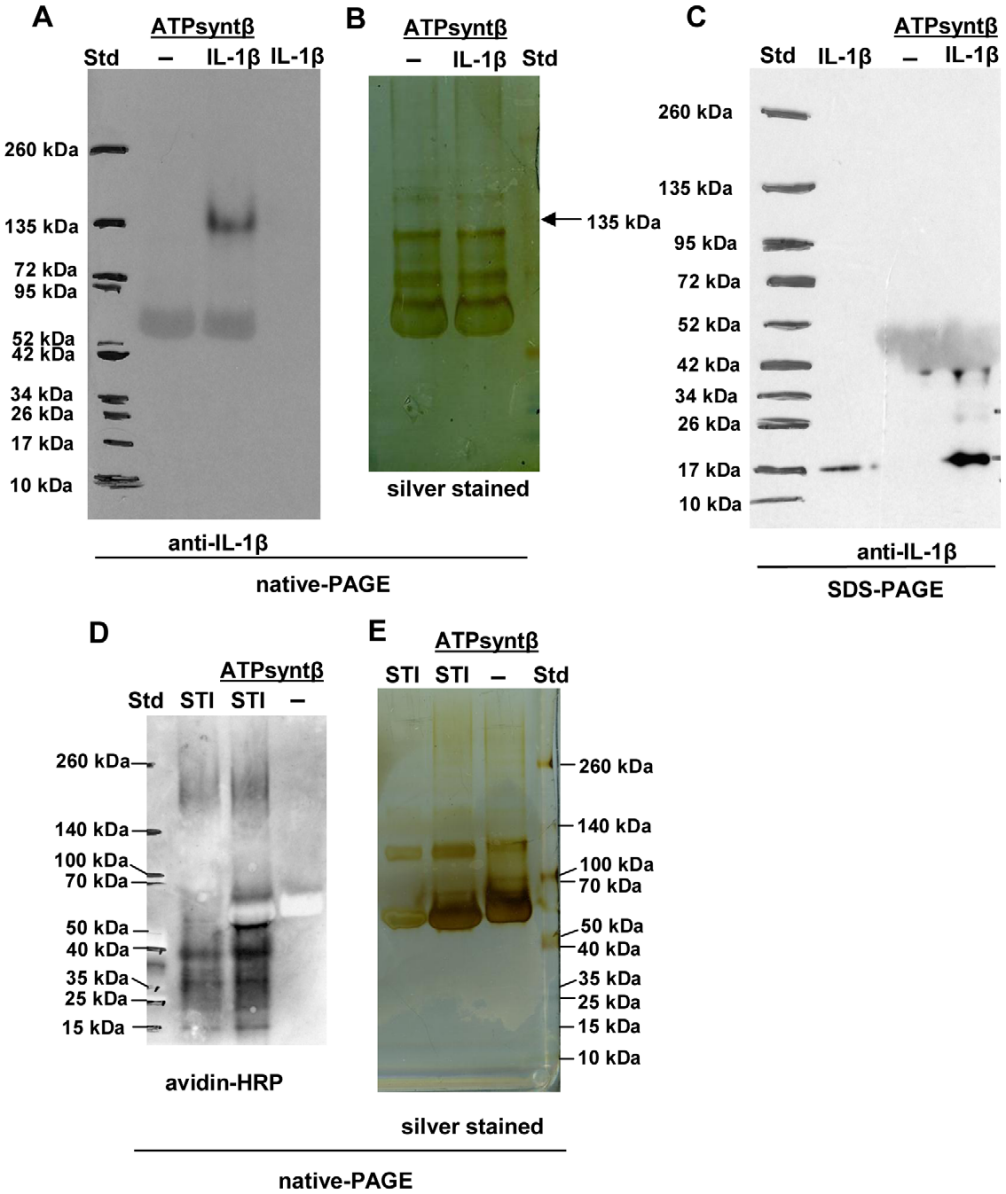
IL-1β binding capacity of recombinant ATP synthase subunit β of *A. actinomycetemcomitans*. Recombinant ATP synthase subunit β (88 µM) was incubated with or without IL-1β (0.29 µM) for 1 h, after which the samples were run in native-PAGE and immunoblotted with anti-IL-1β (Panel A), or silver stained (Panel B). Three prominent forms could be observed from recombinant ATP synthase subunit β (Panel B). IL-1β bound to the trimeric form of recombinant ATP synthase subunit β. IL-1β was not detectable from immunoblotted native-PAGE without pre-incubation with ATP synthase subunit β (Panel A). However, IL-1β was released from the trimeric form of ATP synthase subunit β under denaturing conditions of SDS-PAGE (Panel C). The sizes of ATP synthase subunit β and IL-1β were 51 kDa and 17 kDa, respectively. The binding of the biotinylated control protein soy bean trypsin inhibitor (STI) to ATP synthase subunit β was was estimated similarly (Panel D and E) by using streptavidin-HRP in detection, except the SDS-page was not run. The control protein bound only to the ATP synthase subunit β monomer.

The trimeric form of the recombinant ATP synthase subunit β containing the 8-histidine tag at the C-terminus was needed for binding to IL-1β (Fig. 6A–C). In contrast, the control protein bound to the monomer of ATP synthase subunit β (Fig. 6D and E). According to the ELISA results it seems that ATP synthase subunit β might generally bind more efficiently than, for example, the N-terminal domain of RcpA to the tested small proteins (data not shown). However, IL-1β prefers the trimeric form to other forms of the ATP synthase subunit β.

## Discussion

The aim of this study was to elucidate the mechanisms by which biofilm forming gram-negative bacteria sense IL-1β. IL-1β may be used as a growth promoting factor by fresh clinical isolates of various bacterial species [19]–[21]. IL-1β changes the gene expression pattern of *S. aureus* [4], and IL-1β binding as well as its effects are more pronounced on biofilm cells than on planktonic cells of *S. aureus* [3]. However, any observations about the molecular mechanism that could underlie these phenomena have not been reported. Because IL-1β is produced in the active phase of periodontitis [10], and *A. actinomycetemcomitans* is coupled with aggressive forms of this biofilm disease, we selected *A. actinomycetemcomitans* as a model organism to study the IL-1β – bacterium interaction.

Although enhancing slightly the formation of *A. actinomycetemcomitans* biofilm, IL-1β decreased the metabolic activity of both biofilm and planktonic cells of *A. actinomycetemcomitans*. This decreased activity was dependent on the IL-1β/cell ratio, with a greater ratio producing a longer lasting effect. The metabolic activity indicator, alamarBlue™, is a redox indicator measuring continued growth and cell proliferation. Enhanced biofilm formation of *A. actinomycetemcomitans* support the earlier finding that IL-1β increases the formation of *S. aureus* biofilm [3], but it conflicts slightly with our finding of decreased metabolic activity. It would be logic to assume that enhanced formation of biofilm would be accompanied by increased metabolic activity. Because IL-1β stimulates *S. aureus* to express molecules that recognize adhesive matrix molecules (MSCRAMM) [4] involved in the initiation of biofilm formation, and *A. pleuropneumoniae* — host cell interaction upregulates the expression of genes needed in biofilm formation [11], it could be hypothesized that IL-1β might change the gene expression pattern of *A. actinomycetemcomitans* towards a phenotype with a denser biofilm with reduced metabolic activity. Both of these features might favor the survival of bacteria and resistance to host defense systems *in vivo* [22]. As in *S. aureus*, which lowered the expression of leukotoxins in the presence of IL-1β [4], IL-1β might also regulate the expression of *A. actinomycetemcomitans* leukotoxin, thus having a broader effect on the virulence of the species. However, future research is necessary to establish whether *A. actinomycetemcomitans* responses similarly. The amount of IL-1β used in our experiments (10 ng/ml) lies in the same range as the concentration (up to 80 ng/ml) found in gingival crevicular fluid of active periodontal lesions [10], indicating that the IL-1β effect could have some *in vivo* significance.

Because the specific binding of IL-1β has been reported with various bacterial species [3], [19], [21], we studied the binding of IL-1β on *A. actinomycetemcomitans* clinical strains both in organotypic oral mucosa co-culture and with flow cytometry. The D7S strain biofilm bound strongly IL-1β in the co-culture, and all tested stains bound more IL-1β than control protein on their surfaces as single cells. Although the number of positively stained cells was lower in fimbriae producing rough colony strains than in the non-fimbriated Δ*flp1-flp2* mutant, positively stained rough colony isolates bound more IL-1β per cell than the non-fimbriated control strain. Because fimbriae are the major component of *A. actinomycetemcomitans* that mediates the self-aggregation and adhesion to a variety of surfaces, it could also bind IL-1β. Thus, the observed fluorescence in fimbriated clinical strains, or at least part of it, could originate from IL-1β bound to fimbriae. The finding that the fimbriated strains bound control protein more efficiently than the non-fimbriated strains supports the above mentioned explanation.

It has been hypothesized that extracellular proteases might digest IL-1β into smaller peptides, which could then be transported across the cell membrane and mediate their effects inside the bacterial cell [21]. No clear evidence that this happens has been shown in previous studies, although a potential transfer mechanisms of extracellular signal peptides across the outer membrane of Gram-negative bacteria has recently been reported [23]. The hypothesis is supported by our EM pictures, which show that the antigen IL-1β is attached both inside and outside of the double cell membrane of *A. actinomycetemcomitans* cells that were exposed to IL-1β in a tissue culture model. Moreover, the trimeric form of intracellular ATP synthase subunit β which locates on the cytoplasmic side of the inner membrane, bound to IL-1β. F-ATP synthase produces a majority of the ATP in oxidative phosphorylation and consists of a membrane located F_0_ sector and an intracellular F_1_ sector, which can be solubilized from the F_0_ (for a review, see [24]). The F_1_ sector contains a catalytic domain, which consists of three β subunits [24]. Thus, the finding that the trimeric form of β subunit binds IL-1β is consistent with the finding that IL-1β decreased the metabolic activity of *A. actinomycetemcomitans* cells. Amphibian antimicrobial peptides containing an α-helical structure inhibit *Escherichia coli* ATP synthase by binding to its β subunit, and the degree of inhibition varies between the different peptides [25]. The short extended α-helical loop (QGQDMEQQ) of IL-1β might act similarly once inside the cell. The finding that IL-1β decreased the metabolic activity only transiently might imply that the β subunit has quite a low affinity to IL-1β and that the cells consume the IL-1β quickly. However, in the active phase of periodontitis, IL-1β is produced by the host cells most likely during an extended period, which may simultaneously attenuate the metabolic activity of the *A. actinomycetemcomitans* biofilm.

Because the crosstalk between *A. pleuropneumoniae*, a close relative of *A. actinomycetemcomitans*, and host epithelial cells has been shown to cause the upregulation of biofilm-associated bacterial genes, including secretin coding *rcpA*, which belongs to the *tad*- locus [11], we wanted to investigate if the deletion of some of the components in this Flp-pili producing machinery would affect the ability of *A. actinomycetemcomitans* to respond to IL-1β. Of the tested *tad*-locus mutants, only the Δ*rcpA* mutant was unresponsive to IL-1β. Only one bacterial IL-1β receptor has been identified so far, the Caf1A usher protein of *Yersinia pestis* [26], which is involved in production and secretion of the F1 fimbrial capsule subunit [27]. The *Y. pestis* capsule resembles the *A. actinomycetemcomitans* fimbriae network in appearance [27], [28]. In F1 fimbrial capsule subunit production, the Caf1A usher protein has a similar function to that of RcpA in the Flp1 fimbriae subunit assembly [12], [16], [27]. Therefore, the results obtained from the *tad*-locus mutant strain investigations led us to hypothesize that RcpA could be the IL-1β receptor on *A. actinomycetemcomitans*.

To identify the possible IL-1β receptor, the binding of IL-1β on *A. actinomycetemcomitans tad*-locus mutants was studied using flow cytometry. All tested *A. actinomycetemcomitans tad*-locus mutants had the capacity to bind IL-1β more than control protein. Although the Δ*rcpA* mutant did not respond to IL-1β, it bound biotinylated IL-1β as much or slightly more than the Δ*flp1-flp2* mutant, which does not produce fimbriae but has intact outer membrane Tad machinery. In addition, the recombinant N-terminal domain of RcpA did not bind IL-1β, although it has been suggested to form an Ig-like structure frequently involved in protein-protein and protein-ligand interactions [29]. Of the mutant strains, Δ*rcpA* and Δ*tadD* bound more IL-1β than any other strains. Deletion of a single gene of the *tad*-locus outer membrane protein has been shown to affect the expression levels of other Tad outer membrane proteins [15]. If *rcpA* is deleted, the expression of RcpB decreases moderately and RcpC slightly [15]. Similarly, if *tadD* is deleted, the Δ*tadD* strain produces only subtle amounts of RcpA and RcpB. Integration of the protein expression profiles of different *tad*-locus mutants [15] and our IL-1β binding data suggests that decreased expression of RcpA might lead to increased binding of IL-1β. However, no definite conclusions can be drawn, as the expression data and binding data originate from different mutant clones.

The *in vivo* consequence of the IL-1β binding activity, and of the possible behavioral changes in binding bacterial cells, could mainly be the suppression of local inflammation. Bacterial IL-1β receptors might function similarly to host anti-inflammatory receptors, such as IL-1 decoy receptor (IL-1RII) and single Ig IL-1-related receptor (SIGIRR) (for a review, see [30]), which prevent IL-1β from promoting inflammation. The diminished inflammatory response could therefore protect the bacterial cells. Combined with the decreased metabolic activity, the protection could be double-sided by increasing the bacterial resistance to host defense and by decreasing the inflammatory response aimed at clearing the microbe. Thus, in the progression of chronic infection, the IL-1β binding activity could play a role in the more quiescent phases of disease, or in the phase preceding it. The finding that a conserved intracellular protein interacted with IL-1β implies that ATP synthase subunit β might play a common role in bacterial IL-1β sensing machinery. The next, and probably the most exciting and demanding step, is to identify the specific IL-1β receptor among the outer membrane proteins.

## Materials and Methods

### Bacterial strains and growth conditions

The *A. actinomycetemcomitans* strains used in this study are presented in Table 1. The clinical isolate D7S, which was used as the host strain of the *tad*-locus single gene mutants, originated from a 28-year-old African-American female (USA) with generalized aggressive periodontitis [31]. All *A. actinomycetemcomitans* strains were revived from stocks preserved in 20% skim milk at −80°C, pre-grown on Tryptic soy agar (TSA) supplemented with 5% defibrinated sheep blood, and incubated in candle jars at 37°C for 3 days (rough colony strains) or 2 days (smooth-colony strains) before experiments.

**Table 1:** *Aggregatibacter actinomycetemcomitans* strains used in this study.

Strain	Serotype	Colony morphology	Reference
D7S	a	rough	[31]
SA1398	b	rough	[43]
SA1151	c	rough	[43]
D7SS^a^	a	smooth	[31]
D7SΔ*flp1-flp2* b::Spe^c^	a	smooth	[44]
D7SΔ*rcpA* d::Spe	a	smooth	[44]
D7SΔ*rcpB* e::Spe	a	smooth	[44]
D7SΔ*tadD* f::Spe	a	smooth	[44]
D7SΔ*tadG* g::Spe	a	smooth	[44]

a : spontaneous non-fimbriated variant of D7S;

b : *flp* locus coding for Flp pili subunit Flp1 and a pseudogene *flp2*;

c : spectinomycin resistance cassette;

d : gene coding for outer membrane rough colony protein A;

e : gene coding for outer membrane rough colony protein B;

f : gene coding for outer membrane protein TadD;

g : gene coding for inner membrane protein TadG.

### Formation of *A. actinomycetemcomitans* biofilm in the presence of human IL-1β

Even suspension of plate grown clinical isolates of *A. actinomycetemcomitans* were made according to method by Karched et al. [32] in Trypticase soy broth (TSB) containing 0.6% (wt/vol) yeast extract and 0.8% (wt/vol) glucose (TSB-YE/Glc), and diluted in standard tissue culture treated 48-well cell culture plates (BD Falcon™ #353078, Franklin Lakes, NJ, USA) using TSB-YE/Glc, so that each well contained approximately 5×10^7^ CFU of *A. actinomycetemcomitans*. The cell culture plates were incubated in candle jar for 18 h at 37°C. After pre-growth of 18 h in TSB-YE/Glc, the medium was removed with suction, biofilm was washed with 1 ml sterile 0.85% NaCl, and 950 µl RPMI-1640 medium with glutamine (EuroClone, Milano, Italy) was added. Wells were supplemented with 50 µl recombinant human IL-1β (RELIA*Tech* GmbH, Braunschweig, Germany) to final concentration of 10.0 ng/ml. Sterile water (50 µl) was used as control. Biofilms were incubated in candle jar for 6 h at 37°C before quantification.

Biofilm growth was quantified using method described by Kaplan et al. [33]. Briefly biofilm was stained using crystal violet, washed seven times with 1 ml water, after which the stain was released with 200 µl of 95% ethanol. Half of the total ethanol volume was transferred into wells of 96-well microtiter plate and the A_620nm_ was measured using iEMS microplate reader (Labsystems, Helsinki, Finland). The measured A_620nm_ was related to the one measured from control containing 0 ng/ml human IL-1β.

### Metabolic activity of IL-1β treated *A. actinomycetemcomitans* cells

For biofilm cultivations, even suspensions of plate-grown rough colony isolates of *A. actinomycetemcomitans* were made in TSB-YE/Glc as described above. Suspensions were diluted to 5×10^7^ CFU/ml, or in some experiments to 1×10^7^ or 0.2×10^7^ CFU/ml, in 48-well standard tissue culture-treated plates, so that each well contained approximately 2.5×10^7^ CFU (0.5 or 0.1×10^7^ CFU, respectively, in some experiments). The cell culture plates were incubated in candle jars for 18 h at 37°C. After pre-growth for 18 h in TSB-YE/Glc, the medium was removed, the biofilms were washed with 1 ml sterile 0.85% NaCl, and 475 µl of RPMI-1640 medium with glutamine (Sigma, Steinheim, Germany), but without phenol red. Wells were supplemented with 25 µl recombinant human IL-1β to a final concentration of 10 ng/ml. Sterile water was used as a control. The metabolic activity was measured by adding 50 µl of alamarBlue™ (AbDSerotec, Oxford, UK) to each well and measuring the fluorescence (544 nm excitation, 590 nm emission) of the reduced form of the dye with an Ascent fluorometer (Thermo Labsystems, Helsinki, Finland) at 0, 0.5, 1, 1.5, 2, 3, 4, and 5 h. Biofilms were incubated in candle jars at 37°C between the measurements.

For planktonic cultivations, even suspensions of plate-grown non-fimbriated strains of *A. actinomycetemcomitans* were made in TSB-YE/Glc as described above, and diluted to 1×10^7^ CFU/ml in 14 ml polypropylene round bottom tubes (BD Falcon™ #352006) so that each tube contained approximately 1.0×10^8^ CFU of *A. actinomycetemcomitans*. The culture tubes were incubated in candle jars for 18 h at 37°C. After pre-growth for 18 h in TSB-YE/Glc, a 10-µl sample was taken and plated on TSA-blood plates to confirm that the culture was not contaminated. The cells were washed with 10 ml sterile 0.85% NaCl and suspended in RPMI-1640 medium with glutamine (Sigma) but without phenol red to a concentration of 5.0×10^7^ CFU/ml. An aliquot of 2.4×10^7^ CFU was added to each well of 48-well standard tissue culture-treated plates. Wells were supplemented with 25 µl recombinant human IL-1β to a final concentration of 10 ng/ml, and the metabolic activity was measured by adding 50 µl of alamarBlue™, as described above.

### IL-1β internalization

IL-1β internalization was studied using organotypic oral mucosa [34], [35] — *A. actinomycetemcomitans* biofilm co-culture, which was immunostained with anti-IL-1β IgG after formalin fixation and paraffin sectioning. The stained samples were first visualized under a light microscope, after which positively stained biofilms were examined under a transmission electron microscope.

Briefly, spontaneously immortalized human gingival fibroblasts (HGFs) [36] were suspended in collagen solution (Vitrogen, Cohesion technologies, Palo Alto, CA) mixed with Dulbecco's modified Eagle's medium (DMEM, Life Technologies, Paisley, UK) with 10% fetal calf serum (FCS), 1% essential amino acids and antibiotics (100 µg/ml streptomycin and 100 IU/ml penicillin) at a cell density of 3×10^5^ cells/ml. The cell-collagen suspension (500 µl) was added to each cell culture insert (∅ 10 mm, polycarbonate membrane, pore size 3.0 µm, Nunc, Roskilde, Denmark), placed in the wells of cell culture plates (Costar, Cambridge, MA) and allowed to solidify at 37°C for two hours. FAD medium [DMEM containing 5% FCS, 5 µg/ml insulin (Sigma), 0.4 µg /ml hydrocortisone (Sigma), 5 ng/ml epidermal growth factor (Sigma) and 100 µg/ml ascorbic acid (Merck, Darmstadt, Germany)] was added to the insert and in the wells, and the cell cultures were cultivated for a day. Spontaneously immortalized human gingival keratinocytes (HGKs) [37] were suspended in FAD-medium at a cell density of 3.0×10^5^/ml, and 500 µl of the suspension was seeded on the surface of each collagen-fibroblast gel. Epithelial cells were cultivated one day before the cultures were lifted to the air-liquid interface onto stainless steel grids. Cultures were grown for five days before combining with *A. actinomycetemcomitans* D7S bacteria biofilms.

Even suspensions of the plate-grown *A. actinomycetemcomitans* D7S strain were made in TSB-YE/Glc (containing separately autoclaved glucose) as previously described. A cell suspension containing 5×10^9^ cells in 1 ml TSB-YE/Glc was added to sterile hydrophilic polyethersulfone membranes (5 mm diameter, pore size of 0.2 µm, Supor®-200, Pall Corporation, Ann Arbor, MI) in the bottom of the wells of 48-well cell culture plates, and biofilms were grown in a candle jar at 37°C for 24 hours. Biofilms were briefly washed twice with 0.85% NaCl solution before 24 hours of incubation in RPMI-1640 medium (Sigma) containing 0.6 g/l L-glutamine. Biofilms were co-cultured with organotypic culture model for eight hours.

Formalin-fixed paraffin sections of co-cultures were immunostained with anti-IL-1β using the MonoLink™ Min Polymer Detection System kit (Novocastra Laboratories Ltd, Newcastle Upon Tyne, UK) with a few additional steps. After de-paraffinization, four-micrometer sections were treated with 0.2 mg/ml proteinase K (15 min, 37°C). The endogenous peroxidase block and protein block treatments were done before overnight incubation with rabbit anti-IL-1β antibody (Novus Biologicals, Littleton, CO, USA), control rabbit IgG whole molecule (Jackson ImmunoResearch Laboratories, PA, USA), or control rabbit anti-N-terminal-RcpA antibody (ABCELL, Tampere, Finland) at 4°C. To enhance penetration of the polymer, the sections were treated with post-primary block before adding NovoLinkPolymer. Hydrogen peroxide was used as a substrate and DAB was used as a chromogenic electron donor for the peroxidase reaction, and the sections were counterstained with hematoxylin. For transmission electron microscopy, the sections on slides were fixed with 5% glutaraldehyde in 0.16 M s-collidine buffer. The sections were further fixed in 2% osmium tetroxide in water for 2 h to visualize the DAB-precipitate for electron microscopy, dehydrated and embedded in epoxy resin. The polymerized blocks were removed by heating and cooling from the glass slides, ultrathin sections were cut, picked on grids without further staining [38] and examined with a JEOL JEM-1200EX transmission electron microscope (Japan Electron Optics Laboratory, Tokyo, Japan).

### Binding of biotinylated IL-1β on whole *A. actinomycetemcomitans* cells

The binding of IL-1β on *A. actinomycetemcomitans* cells was studied using the Fluorokine® kit (R&D Systems, Minneapolis, MN, USA). Briefly, biofilm-forming clinical isolates of *A. actinomycetemcomitans* were harvested from culture plates and suspended in TSB-YE/Glc to a concentration of 10^8^ CFU/ml [32], from which 5×10^8^ CFU was taken and cultured in 50 ml tissue culture flasks (Cellstar #690160, Greiner Bio-One, Frickenhausen, Germany) in candle jars for 18 h at 37°C. After overnight cultivation, only flasks with a clear medium were treated as follows. The biofilm was first washed twice with 10 ml Dulbecco's PBS (0.9 mM CaCl_2_, 50 µM MgCl_2_, 2.7 mM KCl, 1.5 mM KH_2_P0_4_, 137 mM NaCl 137, 6.5 mM Na_2_HPO_4_). The biofilm was scraped off and suspended [32], and the cells were fixed with 2.5 ml fixing-solution (1% paraformaldehyde, 1% BSA, 0.01% EDTA in Dulbecco's PBS) for 2 h at 4°C. They were then filtered through a 100 µm Nylon Cell Strainer (BD Falcon™ #2360) to get an even suspension. A total of 1×10^8^ cells were aliquotted, centrifuged (8,000 rpm, 5 min), and suspended in 1 ml of sterile Dulbecco's PBS. Part of this suspension (2.5×10^6^ cells) was mixed with either 10 µl of biotinylated IL-1β or with 10 µl of biotinylated control protein (soybean trypsin inhibitor) having a similar size to IL-1β, and incubated for 1 h at 4°C. After the incubation with biotinylated protein, 10 µl of avidin-FITC (Fluorokine® kit) was added, and the incubation was continued for 30 min at 4°C in the dark. The avidin-FITC-labeled cells were washed twice with 1.5 ml of 1×RDF1 buffer (Fluorokine® kit) and finally suspended in 1 ml of the same buffer. Samples were analyzed with an Epics XL flow cytometer (Coulter Corporation, Miami, Fla.). During flow cytometry, bacterial cells were excited at 488 nm with a 15-mV air-cooled argon ion laser, and the FITC fluorescence was detected through a 525-nm band pass filter. Signals were amplified with the logarithmic mode for side scattering, forward scattering, and FITC fluorescence. Two parameters, mean fluorescence intensity (MFI) and percentage of fluorescence-positive bacterial cells, were determined separately from approximately 5,000 bacteria with a flow rate of 200–300 events/s by gating the bacterial population according to the forward scatter (FSC)/side scatter (SSC) bivariate histogram. To exclude disturbing debris in the FSC/SSC histogram, the discriminant was set to the FSC channel.

Planktonic mutant strains and the spontaneous smooth colony variant of D7S (D7SS) were harvested from culture plates and suspended in TSB-YE/Glc to a concentration of 10^8^ CFU/ml, from which 1.0×10^9^ CFU was cultured as described above. The cells were washed once with 10 ml Dulbecco's PBS, suspended in 2.5 ml fixing-solution, and fixed as described above. The binding of biotinylated IL-1β was then investigated as described above.

### Binding of biotinylated IL-1β on different protein fractions of *A. actinomycetemcomitans* cells

The outer membrane fraction, as well as four distinct protein fractions having different solubilities in the used detergents, were extracted from whole cells of *A. actinomycetemcomitans* D7S [39] and stored at −80°C for further analysis. The total protein concentrations of *A. actinomycetemcomitans* cell fractions were determined using the method of Lowry et al. [40].

The soluble protein fractions were thawed and concentrated using a Vivaspin 2 concentrator with a MWCO of 3 kDa (Sartorius, Goettingen, Germany). Soluble protein fractions (25 µg total protein) were separated by non-denaturing 4–15% tris-HCl precast gel (Criterion, BIO-RAD, USA) and transferred to nitrocellulose membranes (Protran®Whatman®, Dassel, Germany) in an Amersham Biosciences Semi-dry blotter. The membrane was blocked with 5% (w/v) bovine serum albumin (BSA) in PBS (0.01 M Na_2_HPO_4_, 0,15 M NaCl) at pH 7.4 containing 0.1% Tween-20 (Sigma) (PBS-T) at RT for 60 min. After blocking, the membrane was washed with PBS-T at RT and then incubated overnight at 4°C with biotinylated IL-1β (250 ng) (Fluorokine® kit) solution with 0.5% BSA in PBS-T. For the control membrane, a similar blot was prepared using biotinylated soybean trypsin inhibitor (Fluorokine® kit). The membranes were washed with PBS-T before incubation with HRP-linked streptavidin (7 µg) (Sigma) at RT for two hours in 0.5% BSA in PBS-T. The membranes were washed with PBS-T and detected with ECL Western blotting substrate (Pierce®, Thermo Scientific) and Biomax Light film (Kodak, Rochester, NY, USA). The outer membrane protein fraction was not tested, because it was not possible to run it in native-PAGE.

### Identification of IL-1β binding proteins

A non-denaturating 4–15% Tris-HCl precast gel containing 100 µg of total protein was run as described above, and the IL-1β-binding protein bands were isolated from the gel after silver staining [41]. The protein band was oxidized with carbamidomethyl and digested with trypsin before peptide separation with liquid chromatography (C18-colony) and peptide identification with ESI-qTOF (QSTAR Pulsar). The Mascot program combined with the Sprot-Trembl (uniprot) protein sequence database was used for protein identification. The taxonomy was not predefined in order to detect all of the contaminating proteins, including the trypsin used in the digestion.

### Cloning, expression and purification of recombinant ATP synthase subunit β and the N-terminal domain of RcpA, and investigation of their IL-1β binding capacity

The chemicals used were obtained from Sigma-Aldrich (Steinheim, Germany) or Merck (Darmstadt, Germany). PCR primers were obtained from Eurofins MWG Operon (Ebersberg, Germany), and the pET36b vector was obtained from Novagen (Darmstadt, Germany). *E. coli* BL21-CodonPlus(DE3)-RIL cells and *E. coli* XLI blue cells were from Stratagene (La Jolla, CA, USA). The GelJet™ gel extraction kit, plasmid purification kit, and PCR product purification kit were obtained from Fermentas (St. Leon-Rot, Germany).

For cloning procedures, restriction sites (underlined below) were introduced at the 5′end of the respective primers. The forward primer for the ATP synthase β–coding gene, *atpsynβ*, was 5′-ATACATATGGCGACAGGTAAAATTG-3′, and the reverse primer was 5′-ATA CTCGAGCATATTTTTGGCTTTTTCT-3′. For the N-terminal domain (aa: 30–192, excluding the predicted signal sequence) of RcpA, the used forward primer was 5′-ATACATATGCAAAATTTCTCTCTGGATAA-3′, and the reverse primer was 5′-ATACTCGAGAATTTGGGTTGTGTC-3′.

The genes for *atpsynβ* and *rcpA* were amplified by PCR using DNA from *A. actinomycetemcomitans* strains ATCC 700685 (HK1651) and D7S, respectively. Phusion™ High-Fidelity DNA polymerase (Finnzymes, Espoo, Finland) was used in the PCR amplifications. The products of PCR were cloned into pET36b using NdeI and XhoI restriction enzymes, T4 Ligase, and CIAP from Fermentas. The plasmids were transformed into *E. coli* XL1 blue cells by electroporation. The amplified plasmids were sequenced in both directions by Eurofins MWG Operon (Ebersberg, Germany).

The verified plasmid constructs were then transformed into *E. coli* BL21-CodonPlus (DE3)-RIL cells. The transformants were grown at 37°C to an optical density at 600 nm of 1.5 in Terrific broth medium (12 g/l tryptone, 24 g/l yeast extract, 0.4% glycerol, 23.1 g/l KH_2_PO_4_ and 125.4 g/l K_2_HPO_4_) containing 30 µg/ml kanamycin. The expression of proteins was induced for 3 h using 1 mM IPTG (isopropyl β-D-thiogalactoside). Cells were first harvested by centrifugation and then ATPsynβ-containing cells (4.2 g) were resuspended in buffer A (50 mM Na-phosphate, 400 mM NaCl, 20 mM imidazole, pH 7.5) containing 1 mM phenylmethanesulfonylfluoride protease inhibitor and a small amount of DNAseI (Roche Diagnostics GmbH, Mannheim, Germany). They were then sonicated 10 times for 10 s. A cell paste of RcpA protein (4.9 g) was homogenized twice using a french press (SLM Instruments, Urbana, IL, USA).

After centrifugation (at 17,500 rpm for 30 min), the supernatant of ATPsynβ-containing cells was applied to a Ni^+^-charged HisTrap™HP column (Amersham Biosciences), washed with 10% buffer B (50 mM Na-phosphate,400 mM NaCl, 0.5 M imidazole, pH 7.5), and the recombinant proteins were eluted with 30% buffer B. Fractions containing the recombinant protein were pooled and purified further by size-exclusion chromatography on a Superdex 200 26/60 column (GE Healthcare) equilibrated with phosphate buffered saline (PBS) (10 mM Na_2_HPO_4_, 145 mM NaCl, pH 7.2). The fractions containing ATPsynβ were pooled, concentrated and stored at −70°C before use. For the N-terminal domain of RcpA, the purification was done similarly, except Hepes buffer (20 mM Hepes, 100 mM NaCl, pH 7.2) was used in the size-exclusion chromatography. The purity of both proteins was confirmed by SDS-PAGE and Coomassie staining.

For detection of the interaction between IL-1β and ATPsynβ, 96-well plates (NUNC MaxiSorb, Roskilde, Denmark) were first coated with either 100 ng of recombinant human IL-1β (RELIA*Tech*GmbH) or soy bean trypsin inhibitor (Sigma) for three days at 4°C. The dilution was made in PBS containing sodium azide at 0.05% (PSBN). The same volume of PSBN was used as a background control. The wells were washed four times with deionized water. Nonspecific binding sites were blocked by adding blocking buffer [1% (v/v) bovine serum albumin (BSA) diluted in PSB containing 0.05% (v/v) Tween-20 and 0.02% sodium azide (PBS-AT)] and incubating for 2 h at 37°C. After blocking, wells were washed as described above. A serial dilution (98 to 3 µM) was done from recombinant His-tagged ATPsynβ and negative control protein RcpA by diluting the proteins in PBS-AT buffer containing 0.25% BSA. The ELISA procedure for the detection with HisProbe™-HRP (Thermo Scientific, Rockford, USA) was done according to the manufacturer's instructions using ABTS (Sigma) as a peroxidase substrate. ABTS was dissolved to a 1 mM final concentration in 0.01 M sodium citrate buffer at pH 4.2 containing 0.03% H_2_O_2_ (v/v), and the absorbance was measured at 405 nm (iEMS microplate reader, Labsystems, Finland).

For further characterization of the interaction, ATP synthase subunit β (88 µM) and IL-1β (0.29 µM) were incubated in PBS (10 mM Na_2_PHO_4_, 150 mM NaCl, pH 7.5) at RT for one hour. The samples were run on a native page gel, and half of the gel was silver-stained as previously described. Immunoblotting was done to the other side of the gel as a modification of the above-described protocol. After protein transfer, the membrane was blocked with PBS-T (0.05% Tween-20) containing 5% (m/v) milk powder. Overnight incubation was done using 5 µg of rabbit anti-IL-1β antibody (Novus Biologicals) diluted in PBS-T (0.05% Tween-20) containing 0.5% (m/v) milk powder. Amersham ECL anti-rabbit IgG (GE Healthcare) was used in IL-1β detection. The binding experiment with the biotinylated soy bean trypsin inhibitor was done similarly, except the detection was made using 1∶4000 dilution of streptavidin-HRP (Sigma, S2438) according to the manufactures instructions.

### Statistical analysis

The difference between the binding of biotinylated IL-1β and the binding of biotinylated control protein on whole bacterial cells was analyzed using the Wilcoxon Signed Ranks Test. The differences in the binding of IL-1β on various *tad* locus deletion mutants were analyzed using the paired samples T-test. In all statistical analysis, *p*-values<0.05 were considered statistically significant.
